# LuluDB—The Database Created Based on Small RNA, Transcriptome, and Degradome Sequencing Shows the Wide Landscape of Non-coding and Coding RNA in Yellow Lupine (*Lupinus luteus* L.) Flowers and Pods

**DOI:** 10.3389/fgene.2020.00455

**Published:** 2020-05-15

**Authors:** Paulina Glazinska, Milena Kulasek, Wojciech Glinkowski, Marta Wysocka, Jan Grzegorz Kosiński

**Affiliations:** ^1^Department of Plant Physiology and Biotechnology, Faculty of Biological and Veterinary Sciences, Nicolaus Copernicus University, Torun, Poland; ^2^Centre for Modern Interdisciplinary Technologies, Nicolaus Copernicus University, Torun, Poland; ^3^Department of Computational Biology, Faculty of Biology, Institute of Molecular Biology and Biotechnology, Adam Mickiewicz University, Poznan, Poland

**Keywords:** yellow lupine, RNA-Seq, database, flower, pod, miRNA, siRNA, long ncRNA

## Abstract

Yellow lupine (*Lupinus luteus* L.) belongs to a legume family that benefits from symbiosis with nitrogen-fixing bacteria. Its seeds are rich in protein, which makes it a valuable food source for animals and humans. Yellow lupine is also the model plant for basic research on nodulation or abscission of organs. Nevertheless, the knowledge about the molecular regulatory mechanisms of its generative development is still incomplete. The RNA-Seq technique is becoming more prominent in high-throughput identification and expression profiling of both coding and non-coding RNA sequences. However, the huge amount of data generated with this method may discourage other scientific groups from making full use of them. To overcome this inconvenience, we have created a database containing analysis-ready information about non-coding and coding *L. luteus* RNA sequences (LuluDB). LuluDB was created on the basis of RNA-Seq analysis of small RNA, transcriptome, and degradome libraries obtained from yellow lupine cv. Taper flowers, pod walls, and seeds in various stages of development, flower pedicels, and pods undergoing abscission or maintained on the plant. It contains sequences of miRNAs and phased siRNAs identified in *L. luteus*, information about their expression in individual samples, and their target sequences. LuluDB also contains identified lncRNAs and protein-coding RNA sequences with their organ expression and annotations to widely used databases like GO, KEGG, NCBI, Rfam, Pfam, etc. The database also provides sequence homology search by BLAST using, e.g., an unknown sequence as a query. To present the full capabilities offered by our database, we performed a case study concerning transcripts annotated as *DCL 1–4* (*DICER LIKE 1–4*) homologs involved in small non-coding RNA biogenesis and identified miRNAs that most likely regulate *DCL1* and *DCL2* expression in yellow lupine. LuluDB is available at http://luluseqdb.umk.pl/basic/web/index.php.

## Introduction

Yellow lupine (*Lupinus luteus* L.) belongs to a legume family that benefits from symbiosis with nitrogen-fixing bacteria. Seeds of this species are rich in proteins that constitute up to 40% of their dry mass (Ogura et al., 2014). Additionally, years of research and selective breeding have led to the development of alkaloid-free “sweet” cultivars. All these traits make lupine seeds a valuable food source for animals and humans primarily in climatic conditions unfavorable for soybean cultivation (Musco et al., 2017).

The main constraint on a large-scale cultivation of yellow lupine comes from its excessive shedding of generative organs, which contributes to significant yield losses. Therefore, current research focuses on the development of varieties of yellow lupine and cultivation conditions that would prevent massive flower and pod dropping, consequently stabilizing the yield in various environmental conditions (Lucas et al., 2015). Besides its practical significance, yellow lupine is also an excellent model plant for basic research on nodulation (Frankowski et al., 2015) or abscission of generative organs (Glazinska et al., 2017, 2019).

Advances in high-throughput techniques have found new opportunities for deeper exploration of complex nets of factors that regulate biological processes. However, it generates tremendous amount of data, which is impossible to analyze without powerful computers and programming skills. For example, in databases like SRA NCBI, only raw data are deposited, which makes the information unavailable to a wider scientific audience. Due to the current trend in analyzing big amounts of biological data in evolutionary context, it is of great importance to provide the users with the most convenient way possible. One of the best solutions includes the creation of a database with user-friendly interface and downloadable data in the form of analysis-ready tables.

Exemplary databases of this type for other plant species usually contain data on one type of RNA, either encoding proteins (Kawahara et al., 2016; Robinson et al., 2018) or non-coding (Liu et al., 2012; Gupta et al., 2018). Based on their length, non-coding RNAs (ncRNA) are classified into short (<200 nt) and long (over 200 nt) categories (Liu et al., 2015). Short ncRNAs (sncRNA) are represented by miRNA (micro RNAs) and phased siRNA (phased, secondary, small interfering RNAs originally designated as trans-acting small interfering RNAs or ta-siRNAs) (Axtell, 2013a; Fei et al., 2013). They are involved in post-transcriptional control of their target gene activity in the process of RNA interference. Short ncRNAs binding to specific mRNA on the principle of complementarity leads to either its cleavage within the bound sequence or inhibition of its translation (Bartel, 2004; Vaucheret, 2006). Long ncRNAs (lncRNAs) were shown to be potent *cis*- and *trans*-regulators of gene transcription, and can act as (i) scaffolds for chromatin-modifying complexes, (ii) decoy for splicing factors, or (iii) competitors for miRNA binding sites (Marchese et al., 2017). Regarding legumes, a LegumeIP 2.0 database is available, containing data on genomic and protein-coding sequences for six legume species: *Medicago truncatula* (lucerne), *Glycine max* (soybean), *Lotus japonicus* (birdsfoot trefoil), *Phaseolus vulgaris* (common bean), *Cicer arietinum* (chickpea), and *Cajanus cajan* (pigeon pea) and two outgroup reference species: *Arabidopsis thaliana* and *Populus trichocarpa* (Li et al., 2016). Other examples are SoyNET (Kim et al., 2017) and SoyKB (Joshi et al., 2017) for soybean. The latter is the most extensive one, which also contains information on sRNA sequences, however, only for *Glycine*.

In case of *Lupinus luteus* database (LuluDB), our aim was an integration of our RNA-Seq data for yellow lupine protein-coding RNA and ncRNA sequences in one place. Besides protein-coding transcripts, LuluDB contains information about known and novel miRNAs, siRNAs and their target transcripts, as well as lncRNAs. LuluDB provides information about sequences, their accumulation in generative organs and identified target transcripts for sRNAs. It is possible to view the database by scrolling the interactive list of elements, search it by sequence ID, or query it by homological sequence using built-in BLASTn. LuluDB will popularize the genetic research on this important crop plant and support the research on universal regulatory mechanisms of plant development mediated by ncRNAs.

Detailed analysis of the data concerning miRNA and siRNA in yellow lupine flowers has been already published (Glazinska et al., 2019), while similar analysis conducted for pods is presented here for the first time. Similarly, data on putative lncRNA in lupine have not been published yet. A short presentation of these results will be presented here together with a description of the capabilities of the database. In addition, to prove the usefulness of the database, we present a case study of transcripts identified as *DCL1–4* (*DICER LIKE 1–4*) homologs involved in the small ncRNA biogenesis process (Fukudome and Fukuhara, 2017) and identified miRNAs that most likely regulate *DCL1* and *DCL2* expression in yellow lupine. The database is available at http://luluseqdb.umk.pl/basic/web/index.php.

## Description of Utility and Discussion

### Data Sources and Generation in LuluDB

LuluDB was created on the basis of NGS sequencing analysis of sRNA, transcriptomes, and degradome libraries obtained from generative organs of yellow lupine cv. Taper: flowers in various stages of development, developing pod walls and seeds, flower pedicels, and pods undergoing abscission and control ones. Through this experimental design, we aimed at examining global changes in expression during flower development, and wanted to determine the differences in their development depending on the location in the inflorescence, which is associated with the tendency to fall off/transform into pods (van Steveninck, 1959). Therefore, flowers from the highest whorls of inflorescences (of which 100% undergo abscission) (UF) and from the lowest (100% binds pods) (LF) were collected separately in four developmental variants. The first stage consisted of closed, yellowing, elongating buds with closed anthers, and the second stage consisted of closed buds with yellow petals and open anthers; during the third stage, there were flowers in full anthesis, with visible pollen on the stigma, and the fourth stage comprised open flowers with aging anthers, and no trace of sticky pollen, but with yellow petals retaining turgor. The ovules of the lower fourth stage flowers were enlarged. To explore the landscape of RNA expression during the abscission process, we also collected pedicels of flowers at stage 3 from the lower whorls (FPNAB) and of flowers with symptoms of abscission and senescence (FPAB). Due to the fact that lupine pods fall off at the initial stages of development, we also sampled young green pods without active abscission zone (PNAB) and pods of the same age with visible symptoms of abscission (PAB). Yellow lupine is a plant valued for its seeds (Lucas et al., 2015). Understanding the global changes in gene expression during the development of pods of this plant may become a valuable contribution to various studies aiming at improving crop yields. The pod RNA libraries were constructed separately from seeds (S) and pod walls (W) collected in eight time points, and then combined into three variants, where the first one (P1) included early developmental stages characterized by intensive growth, the second one (P2) wherein seed filling occurred, and the third one (P3) where the filling ended and the pods started to ripen and desiccate. The detailed list of samples is presented in the Table 1.

**Table 1:** List of samples deposited to date in the LuluDB database.

**Alias**	**Sample name**	**Description**	**RNA-Seq**	**Small RNA-Seq**	**Degradome**	**References**
UF1	Upper flowers stage 1	Flowers from upper part of raceme in stage 1	•	•		(Glazinska et al., 2019), this study
UF2	Upper flowers stage 2	Flowers from upper part of raceme in stage 2	•	•		(Glazinska et al., 2019), this study
UF3	Upper flowers stage 3	Flowers from upper part of raceme in stage 3	•	•	•	(Glazinska et al., 2019), this study
UF4	Upper flowers stage 4	Flowers from upper part of raceme in stage 4	•	•		(Glazinska et al., 2019), this study
LF1	Lower flowers stage 1	Flowers from lower part of raceme in stage 1	•	•		(Glazinska et al., 2019), this study
LF2	Lower flowers stage 2	Flowers from lower part of raceme in stage 2	•	•		(Glazinska et al., 2019), this study
LF3	Lower flowers stage 3	Flowers from lower part of raceme in stage 3	•	•	•	(Glazinska et al., 2019), this study
LF4	Lower flowers stage 4	Flowers from lower part of raceme in stage 4	•	•		(Glazinska et al., 2019), this study
FPNAB	Flower pedicels non-abscissing	Pedicels of non-abscissing flowers	•	•		(Glazinska et al., 2019), this study
FPAB	Flower pedicels abscissing	Pedicels of abscissing flowers	•	•		(Glazinska et al., 2019), this study
PW1	Pod walls stage 1	Pod walls in early stage of development	•	•		This study
PW2	Pod walls stage 2	Pod walls in middle stage of development	•	•		This study
PW3	Pod walls stage 3	Pod walls in late stage of development	•	•	•	This study
PS1	Pod seeds stage 1	Seeds in early stage of development	•	•		This study
PS2	Pod seeds stage 2	Seeds in middle stage of development	•	•		This study
PS3	Pod seeds stage 3	Seeds in late stage of development	•	•	•	This study
PNAB	Pods non-abscissing	Non-abscissing pods	•	•		This study
PAB	Pods abscised	Abscissing pods	•	•		This study

After the sequencing and preliminary data analysis, the data concerning sequences of identified coding RNAs and ncRNAs were first deposited in the raw form in NCBI SRA database and then analysis-ready data were uploaded into the LuluDB database. Table 1 and Supplementary Table 1 present details of the data deposited in NCBI SRA and the LuluDB.

### ncRNAs in LuluDB

The database contains sequences of 456 known and 32 novel miRNAs, as well as 318 phased siRNAs identified in yellow lupine along with information about their expression and target transcripts. In our previous paper (Glazinska et al., 2019) in which part of the data described here supported the evidence that sRNAs are involved in yellow lupine flower development and abscission, each miRNA received a unique ID number on a slightly different principle than it is presented in LuluDB. Namely, known miRNAs [i.e., having identical hits in miRBase (Kozomara et al., 2019)] were assigned IDs from Ll-miR1 to Ll-miR456, and the numbering of novel miRNAs [identified with ShortStack (Axtell, 2013b)] started from the beginning, with the “n” inset before the number (e.g., Ll-miRn22). As the small RNA-Seq technology becomes more available, we predict an increase in deposition of new miRNA sequences to miRBase, and in the future, it might be discovered that some of the lupine miRNAs currently considered as novel have homologs in other plant species, and such numeration would be misleading. This is why in LuluDB the numbering within novel miRNA IDs continues after the last known one, from 457 up. Besides the identification of novel miRNAs, ShortStack was used to identify small RNA cut in phase from longer precursors (phased siRNA).

Expression of small RNAs in individual samples is stated in RPM (reads per million). For both miRNAs and siRNAs, potential target transcripts were identified by degradome data analysis carried out with CleaveLand4 (Addo-Quaye et al., 2009) and by additional *in silico* analysis with psRNATarget toolkit (Dai et al., 2018) in order to predict targets that are not only cleaved, but may also be suppressed in other modes of sRNA action.

We have identified lncRNAs by performing BLASTn search within CantataDB (Szcześniak et al., 2019) in which *G. max* lncRNAs were queried by transcripts obtained in our experiment. As a result, 31,718 lncRNA sequences homologous to those in soybean were found and deposited in LuluDB.

### Coding RNAs in LuluDB

LuluDB contains 267,349 protein-coding RNA sequences with annotations to commonly used databases: Blastp, Blastx, Eggnog, KEGG, CantataDB, miRBase, NCBI protein, Pfam, Rfam, and GO. Because the reference yellow lupine genome sequencing is still in progress (Iqbal et al., 2020), the transcripts were assembled *de novo*. This assembly was carried out separately for libraries derived from flowers (already published in Glazinska et al., 2019) and pods with Trinity toolkit, which assigned an ID for each transcript (e.g., TRINITY_DN10038_c0_g1_i1) within each batch separately. This created the risk that completely dissimilar transcripts in flowers and pods could have the same ID. We fixed this in three ways: (i) by providing each transcript with information about its origin (flowers or pods), (ii) by adding the “F” prefix to TRINITY ID in flower dataset (e.g., FTRINITY_DN53848_c2_g1_i5), and (iii) by assigning additional ID for the database (e.g., LI_transcript_534367). All of the assembled transcripts were clustered and, within each cluster, they were assigned “Gene” name: (e.g., LI_gene_11901). Analysis indicates that the majority, but not all of the transcripts, have their near-identical homologs in both types of organs (Supplementary Figure 1). Minor differences may be caused either by assembly errors or the specificity of transcript processing (such as alternative splicing).

The expression levels in FPKM unit (fragments per kilobase of exon per million fragments mapped) are shown only for the relevant libraries.

### Database Organization

**Figure 1:**
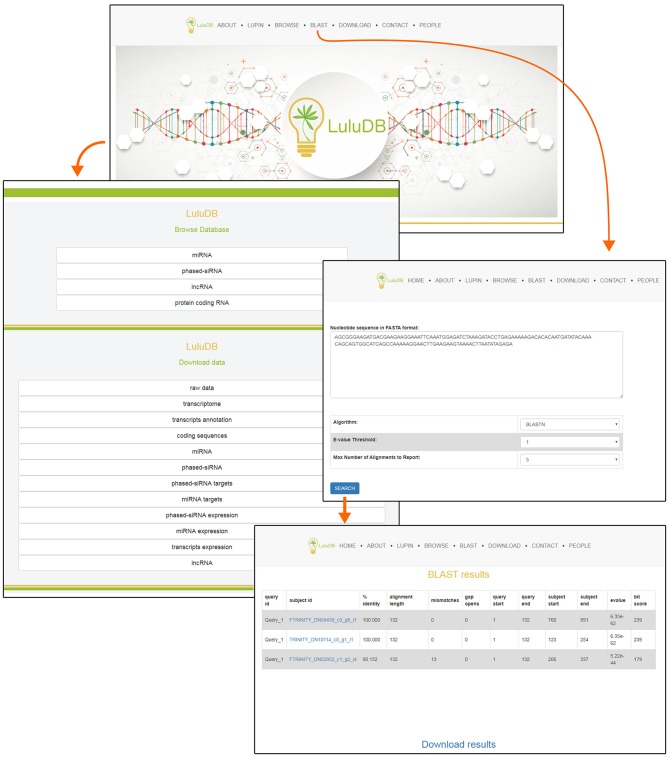
Screenshot of LuluDB home page and of the interface to submit BLAST searches.

You can easily navigate to major database components from the top of the home page (Figure 1). The selection includes general information (About), information on *L. luteus* (Lupin), browsers for various types of deposited sequences (Browse), integrated BLAST search tool for user sequences (BLAST), data download links (Download), contact information to corresponding author (Contact), and information on everyone involved in the creation of LuluDB (People) (Figure 1).

**Figure 2:**
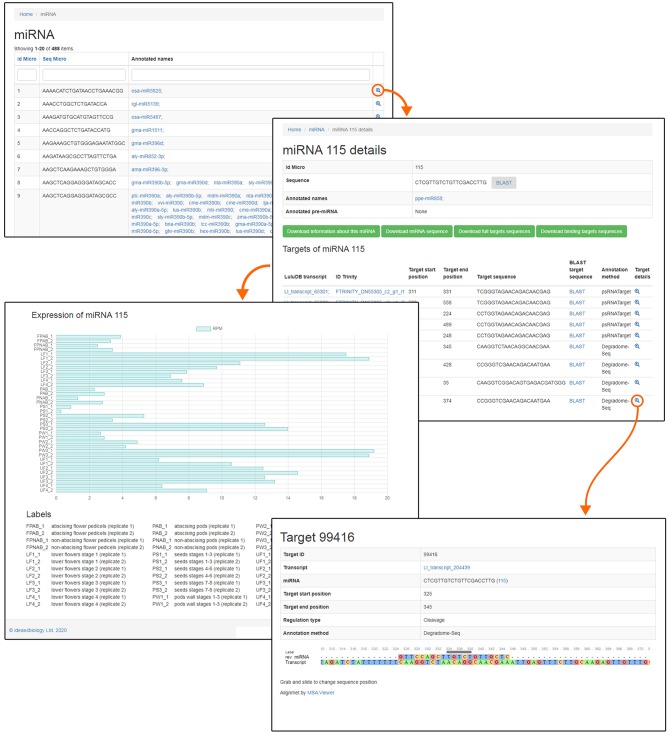
Screenshot of LuluDB page concerning example miRNA.

One of the most crucial elements of the home page is the Browse section. This page contains links to various parts of the database, such as miRNA, phased siRNA, lncRNA, as well as protein-coding RNAs (Figure 1). A list of identified miRNAs can be found in the miRNA section and searched by sequence ID, RNA sequence, or miRBase annotation (Figure 2), and the annotations also serve as links to relevant miRBase entries. The magnifying glass icon in the last column leads to the details site where you can find information about its Id Micro, Sequence, Annotated names, and Annotated pre-miRNA. If target transcripts were identified for this particular miRNA, a handy table containing detailed information about it is displayed lower in this page. Below, expression of the miRNA is plotted for each library as a bar plot. It is possible to download all data contained on this page by clicking clearly described buttons. In the “details” section, the BLASTn tool (redirect to BLASTn with already loaded sequence of either miRNA and target gene) allows for a quick analysis.

The phased siRNA section is structured in similar manner (Supplementary Figure 2A).

On the lncRNA main page, there is a list of transcripts identified as lncRNAs, composed of ID from Cantata, LuluDB transcript ID, and Trinity Id (Supplementary Figure 2B). Clicking the magnifying glass icon in the last column leads to a page with details on the given transcript: internal LuluDB ID, ID from Cantata with hyperlink to that database, LuluDB transcript ID with internal hyperlink to more details about this transcript, including its expression, and its sequence. As in previous cases, all this information can be downloaded. This part of the database is the least extensive due to the limited ways of analyzing lncRNAs.

In the protein-coding transcript section, the list of transcripts can be searched by TRINITY ID, LuluDB ID, or ORF type, which can be: “complete,” “internal,” “3prime_partial,” or “5prime_partial” or annotations to various databases (Supplementary Figure 3). Under the magnifying glass icon, there is a link to a page with details: LuluDB ID, CDS coordinates, predicted amino acid sequence, and details about annotation to following databases: Blastp, Blastx, Eggnog, Kegg, CantataDB, miRBase, NCBI protein, Pfam, Rfam, GO, and graphical representation of ORF. All of the data are available for download. Under LuluDB ID, there is a hyperlink to page with more information about this transcript, including its expression and annotation details. Table 2 illustrates the number of transcripts identified in yellow lupine annotated to public databases.

**Table 2:** Summary of protein-coding transcripts deposited to date in LuluDB annotated in various open access databases.

**Public database**	**No. of annotated unigenes**
RFAM	4,433
PFAM	198,225
CantataDB	31,718
Nr	288,854
SwissProt	216,711
KEGG	247,375
GO	534,413
miRBase	2,565

### Validation of sRNA and RNA Sequencing Results Deposited in LuluDB

**Figure 3:**
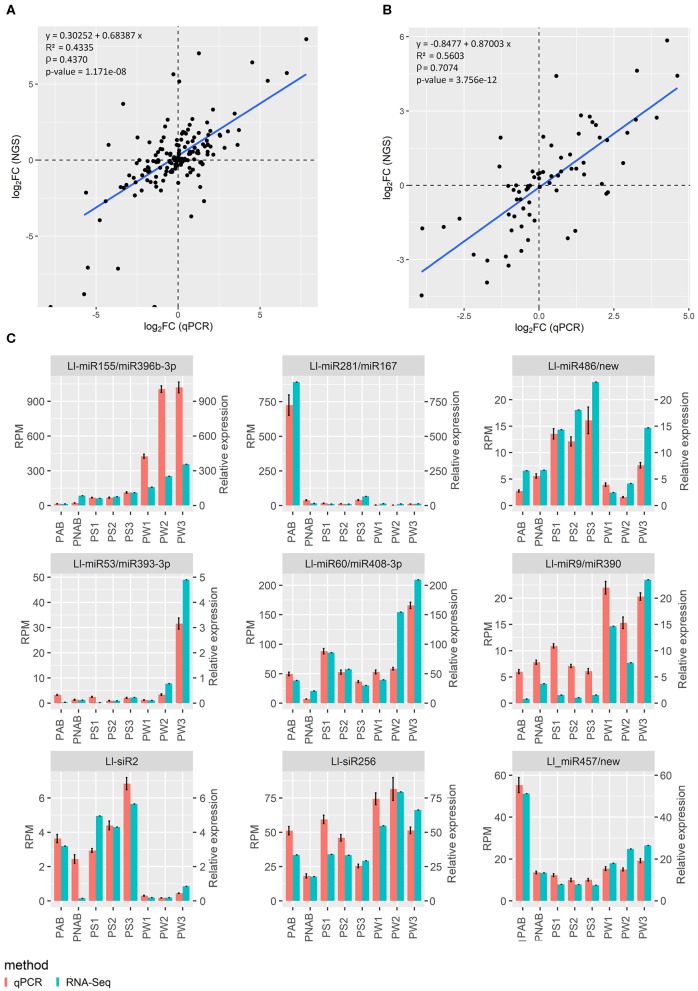
Relationship between next-generation sequencing and qPCR results. **(A)** Log_2_ fold change of gene expression assessed using NGS plotted against log_2_ fold change of gene expression assessed using qPCR. **(B)** The same plot for sRNA data. *R*^2^ is coefficient of determination, ρ is Spearman's rank correlation coefficient. **(C)** Graphs showing the similar trend in expression levels of miRNAs and siRNAs assessed with NGS and qPCR.

Results of sRNA and RNA deep sequencing were validated using qPCR technique. Validation for data concerning flowers was already presented in our recent paper (Glazinska et al., 2019) and the same approach was used to validate the rest of the results. Regarding sRNA-Seq, 7 miRNA, and 2 siRNA sequences were selected and subjected to stem-loop RT-qPCR technique (Kramer, 2011; Varkonyi-Gasic and Hellens, 2011), while in the case of RNA-Seq, the standard qPCR reaction was carried out for eight transcripts. Figure 3 shows the plotted log_2_ ratio of fold changes (FC) calculated from qPCR against log_2_ FC of the sRNA-Seq (Figure 3A) or RNA-Seq data (Figure 3B). Figure 3C shows the validation data for nine abovementioned small RNAs and Supplementary Figure 4 displays the validation data of eight transcripts used for validation for homologous transcripts found in both flowers and pods. The *R*^2^ and Spearman's rank correlation coefficient (rho) are satisfactory and confirm the linearity of the relationship between qPCR and sRNA-Seq or RNA-Seq data (Figures 3A,B), and similarity in expression patterns measured with NGS and qPCR (Figure 3C and Supplementary Figure 4) supports the validity of our RNA-Seq.

## Case Study

### Identification of Homologs of DCL Family Members in Yellow Lupine

In order to show the functionality of LuluDB and present an exemplary analysis pipeline, we used the database interface to identify transcripts encoding homologs of *A. thaliana* DCL1, 2, 3, and 4 involved in small ncRNA biogenesis (Fukudome and Fukuhara, 2017) and miRNAs that post-transcriptionally regulate their expression in yellow lupine.

Firstly, we downloaded the CDS sequences of selected *A. thaliana DCL* genes from NCBI (NM_001197952, NM_001202869.2, NM_001161191.3, NM_001203419.2) (Supplementary Table 2). Then, we queried LuluDB with these sequences using local BLASTn tool with the following parameters: *E*-value Threshold: 1e^−10^ and Max Number of Alignments to Report: 100. For respective DCL sequences, different lists of results were acquired (Supplementary Tables 3–6 for DCL1, 2, 3, and 4, respectively). It is noteworthy that our database allows the users to download BLASTn results. The most homologous sequences were found for *DCL2* and the least were found for *DCL1* and *4*, and in each case, it was found in both tissues, flowers and pods. The pages for individual transcripts contain detailed information, such as ID, sequence, annotation, and expression in different organs, which can be viewed and downloaded, and the “details” link redirects the user to amino acid sequence and additional information. We employed two ways of obtaining the data: manual, where we enter the site of each identified transcript one by one and download all available information, or more automated, where we use Python script to search the files downloaded from the main page with the lists of homologous transcripts from BLASTn. Both ways gave the same results.

All sequences identified by BLASTn are annotated as *DCL* genes (Supplementary Tables 3–6). Only the transcripts with complete ORFs and coding the same protein in both tissues, flowers and pods, were used for further analysis. The only exception here is *DCL2*, where the only transcript in flowers with complete ORF has no equivalent in the set of transcripts in pods, which are much shorter.

In *A. thaliana*, four members of DICER-like family are responsible for sncRNA biogenesis of different lengths (Gasciolli et al., 2005). DCL1 is involved mainly in biogenesis of 21 nt miRNA by cutting out miRNA/miRNA^*^ duplexes from imperfect fold back stem-loop structures within pri-miRNA and pre-miRNA precursors (Liu et al., 2005; Song et al., 2011). DCL2, DCL3, and DCL4 are responsible for generating siRNA from dsRNA derived from exogenous elements, natural antisense genes, transcripts of *TAS* genes, and probably *PHAS* genes, or repeated heterochromatic regions (Gasciolli et al., 2005; Henderson et al., 2006; Rajagopalan et al., 2006; Chen et al., 2010). Despite the differences, functions of all DCL enzymes partly overlap (Gasciolli et al., 2005).

In many plant species *DCL* genes are duplicated. For example, rice genome encodes two isoforms of *DCL2* and two of *DCL3* (Kapoor et al., 2008). Similar situation occurs in legumes. In soybean, seven *DCL* genes were described, two isoforms of *DCL1, DCL2*, and *DCL4* each and one of *DCL3* (Curtin et al., 2012; Liu et al., 2014b). In *M. truncatula* genome, six *DCL* genes encode members of all four types of DCL (DCL1–4), including three isoforms of DCL2 (Tworak et al., 2016). In *L. japonicus*, five subsequent *LjDCL* genes were identified: *LjDCL1, LjDCL2a* and *LjDCL2b, LjDCL3*, and *LjDCL4* (Bustos-Sanmamed et al., 2013). Within narrow-leaved lupine genome, seven *DCL* genes were found, including three homologs of *DCL2*, two of *DCL3*, and one of *DCL1* and *DCL4* (DeBoer et al., 2019).

**Figure 4:**
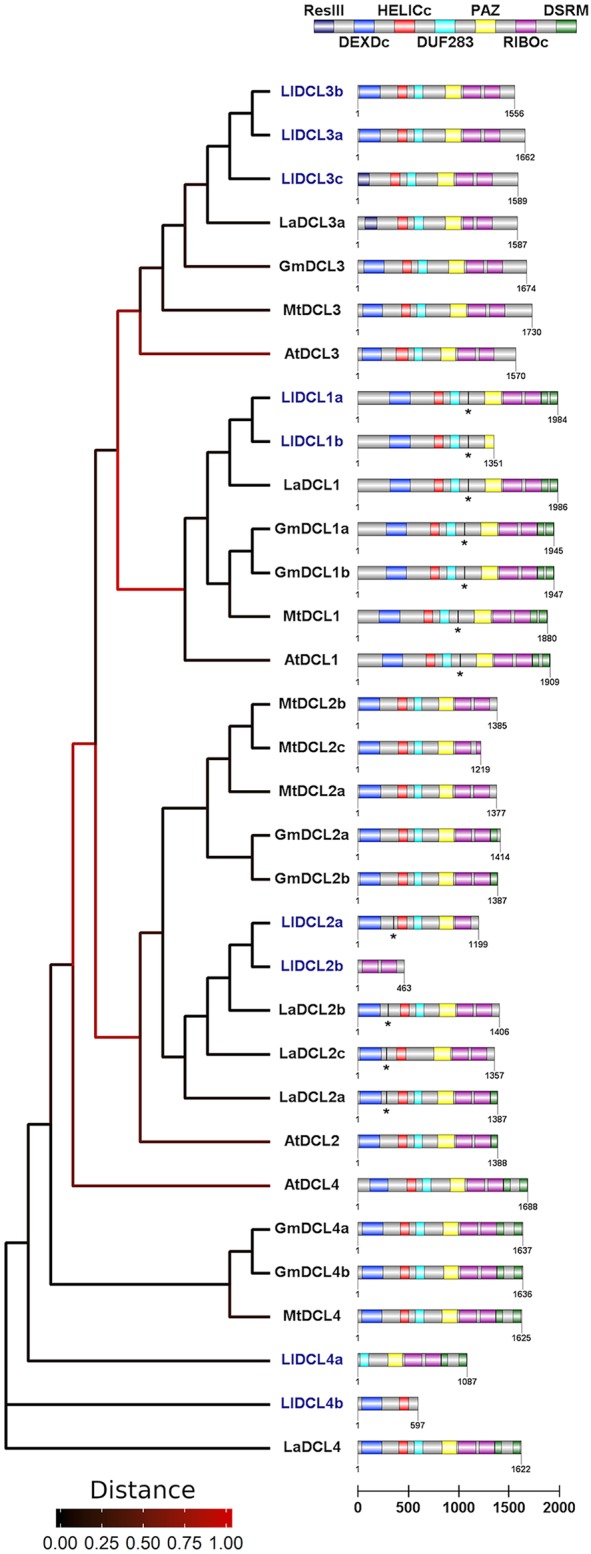
Phylogenetic tree and domain structure of members of DCL families identified in *Lupinus luteus* (Ll), *Arabidopsis thaliana* (At), *Medicago truncatula* (Mt), *Glycine max* (Gm), and *Lupinus angustifolius* (La). Asterisks indicate predicted miRNA target sites. Lupine sequences were translated using SeqIO biopython package, the phylogenetic tree was created using *Phylogeny.fr* interface, and the domain architecture was drawn using DOG v 2.0 software.

In this work, we have demonstrated that members of all *DCL* families are present and expressed in generative organs in yellow lupine. Our results suggest the presence of more than one gene that codes for each type of DCL protein, similar to other *Fabaceae* (Figure 4). On the basis of both nucleotide and amino acid sequences, we were able to distinguish two isoforms of *DCL1, DCL2*, and *DCL4*, and three of *DCL3*, all of which show greatest similarity to the corresponding homologs *Lupinus angustifolius* (Figure 4). Please note that these results do not show the total number of *DCL* genes in *L. luteus*; they only show which of them are expressed in generative organs. In order to reveal the complete landscape of *DCLs* in yellow lupine, we need to analyze the sequence of its genome, which is still unavailable (Iqbal et al., 2020).

In plants, members of DCL protein families contain six conserved domains: N-terminal helicase domain (built with DEXD/H-box and helicase-C subdomains), followed by DUF283 (domain of unknown function, also known as Dicer-dimer or Dicer dimerization domain), PAZ (Piwi-Argonaute-Zwille), tandemly arranged two RNase III domains, and up to two C-terminal dsRBD (dsRNA binding) domains (Carmell and Hannon, 2004; Margis et al., 2006; Murphy et al., 2008). The main catalytic activity is demonstrated by two RNase III domains, which cleave dsRNA substrates and form short RNA duplexes. PAZ and helicase domains are known to play a role in proper docking of sncRNA precursor within DCL protein (MacRae et al., 2006; Gu et al., 2012), and helicase additionally enables processing of longer substrates (Cenik et al., 2011; Welker et al., 2011). The dsRBD domain is thought to be involved in the recognition and processing of RNA substrates as well as in interactions with other elements of sncRNA biogenesis pathway (Hiraguri et al., 2005; Eamens et al., 2009).

The presence of all of the abovementioned domains in DCL1 is highly conserved across plants including legumes, which proves that it plays the most important role in sncRNA biogenesis (Gasciolli et al., 2005; Parent et al., 2012). The domainal organization of other DCL proteins is more varied in different plant species, which is probably related to their overlapping functions and the resulting increased tolerance to aberrations.

In our analyses, only the *DCL1* transcript encodes protein sequence containing all possible DCL domains (Figure 4). In the other cases, the putative proteins are truncated and lack either C- or N-terminal domains. Regarding DCL2 and DCL3, the C-terminal truncation seems to be evolutionarily conserved, and these proteins contain at most one single copy of the DSRM domain, and in the case of MtDCL2c and LlDCL2b—they also lack one of the RIBOc domains. In the set of studied DCL2 homologs, only LlDCL2b is truncated at the N-terminus, and as it lacks all the domains except for two RIBOc, it is probably a pseudogene. In LlDCL3c and LaDCL3a, the N-terminal domain DEXDc is narrowed to sequence encoding the res subunit of type III restrictase (ResIII). It is striking that if we merged LlDCL2a and b, as well as LlDCL4b and a, we would obtain a full-length protein. This fact suggests that some mutations in *DCL2* and *4* homologs in yellow lupine might have occurred, leading to the emergence of non-functional or partly functional proteins. Thus, problems with identifying full-length ORF sequences of *DCL2* in pods may arise because of similar reasons: mutations that change the reading frame or influence alternative splicing, or other unknown causes. It is difficult to determine the physiological effects of the presence of truncated DCL proteins in yellow lupine; however, considering that we have identified a number of siRNAs, e.g., tasiR-ARF (Glazinska et al., 2019), the biogenesis of siRNA in this plant is unaffected. Perhaps the truncated proteins form complexes with more complete DCLs, which enables their participation in sncRNA biogenesis. It would be interesting to examine the exact function of these DCLs.

In addition to analyzing the putative amino acid sequence of DCLs, we have also explored nucleotide sequences of transcripts, which encode these proteins. The mRNA and CDS sequences deposited in the database enable the identification of non-translated sequences, e.g., 5′UTR, which often contain regulatory sequences providing premises for speculation on the possible factors affecting expression of the studied genes. We performed sequence analysis of 5′UTR regions of genes encoding DCL1, 3, and 4 (Supplementary Table 7), except for *DCL2*—because of its shortness (~80 nt). In the case of *DCL4*, the mRNA sequence upstream to the origin of the identified ORF is as long as 1,726 nt, which is an extremely long 5′UTR. This may be associated with the N-terminal truncation of DCL4 protein, probably caused by a mutation that either turned off the original start codon and switched the start of translation downstream to the next ATG, or turned on the stop codon between original start codon and the next ATG. Further analyses support the latter hypothesis, as in FTRINITY_DN57273_c0_g1_i5 transcript; for example, there is an additional ATG at 173 nt located farther than in *A. thaliana*, which may encode a protein containing missing N-terminal domains, but it is stopped prematurely. Moreover, these two ORFs are placed in different reading frames. In the LuluDB, only the longest CDS identified on a given mRNA was deposited. For 5′UTRs of *DCL1* and *3*, they are 604 and 201 nt long, respectively.

We have analyzed selected 5′UTRs by querying PlantCare, a database of plant *cis*-acting regulatory elements and a portal to tools for *in silico* analysis of promoter sequences (Lescot, 2002). All UTR sequences contain typical promoter elements, namely, CAAT (common *cis*-acting element in promoter and enhancer regions) and TATA box (core promoter element around −30 of transcription start) as well as elements related to the response to plant hormones (e.g., IAA, SA, or ABA) or stress factors and light (Supplementary Table 7). This demonstrates the complex regulation of expression of these transcripts.

**Figure 5:**
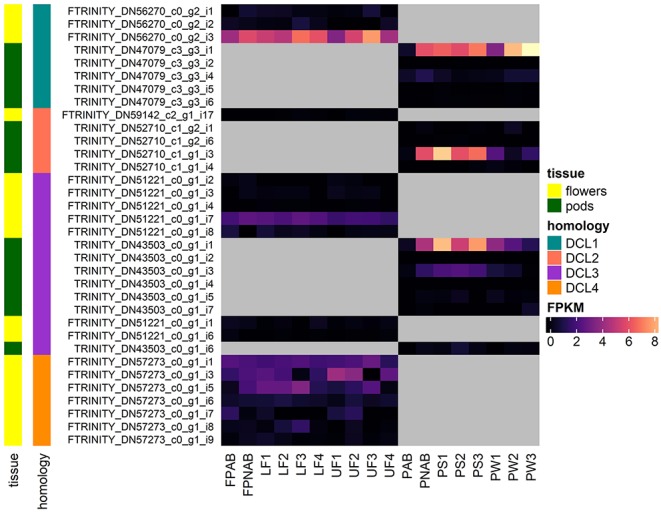
Heatmap presenting expression of RNAs coding for DCLs identified in yellow lupine, created using the “ComplexHeatmap” R package.

Figure 5 shows the expression of all LuluDB BLASTn hits containing complete ORFs. The overwhelming majority of them show low or even extremely low expression in most of the tested samples. One exception is mRNA encoding DCL1a in both flowers and pods, which exhibits the highest level of expression in stage 3 of flower development, and in the oldest walls and seeds of pods. Other exceptions include single transcripts encoding DCL2 and 3, which accumulate the most in yellow lupine pods. Interestingly, shortened transcripts (containing partial ORFs) of *DCL2* and *DCL4* have higher accumulation (Supplementary Tables 4, 6). It would be extremely interesting to investigate the cause. Our hypothesis, which says that they are products of cutting longer transcripts, requires further analysis.

In soybean, *DCL2* expression is regulated by miR1515 (Li et al., 2010), and in *Medicago* by miR1507 (Zhai et al., 2011). Our analysis shows that in yellow lupine, none of these miRNAs targets any of DCL2-encoding transcripts: miR1515 is missing from our libraries, as well as the target transcripts of miRNA annotated as miR1507 (ID88) code for members of the Disease resistance protein RGA family. Exploring the data deposited in LuluDB can lead to further research opportunities. Our short case study on *DCL* genes, for example, evoked several questions and presented new exciting challenges, including an analysis of changes in the expression of identified genes under the influence of factors predicted by 5′UTRs analysis, or complete characteristics of genes encoding DCLs as soon as good quality genome of *L. luteus* is released.

### Analysis of miRNAs That Target *DCL1* and *DCL2* in Yellow Lupine

Literature data contain evidence that miR162 regulates the *DCL1* expression in other plants (Xie et al., 2003; Liu et al., 2014a; Szajko et al., 2019), whereas in yellow lupine, we have identified a new regulator of *DCL2* (Glazinska et al., 2019), a novel miRNA named “Ll-miRn30,” deposited in the database under ID486.

**Figure 6:**
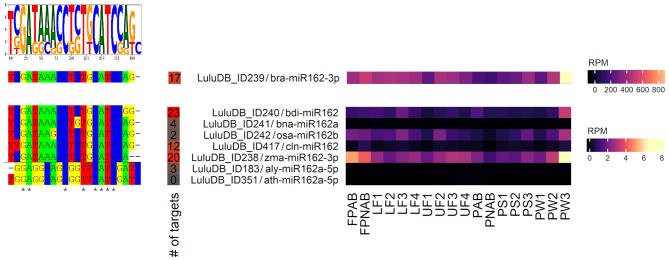
Homologs of miR162 identified in yellow lupine. Left: aligned miRNA sequences with the sequence logo on the top. Right: Heatmap presenting expression of homologs of miR162 identified in yellow lupine, created using the “ComplexHeatmap” R package.

After typing the phrase “miR162” in the search bar of the browse/miRNA section, we are presented with a list of eight lupine miRNAs annotated as miR162, which means that they are identical to miR162s from other plant species deposited in miRBase (Figure 6). This indicates that sequences of lupine miR162 are evolutionarily conserved; miRNA of ID239 is most conserved and ubiquitous, as it is annotated to the biggest number of miRNA sequences from miRBase (Kozomara et al., 2019).

*L. luteus* miR162s are annotated to both miR162-3′ and miR162-5′. The detailed analysis showed that five of them (ID238, 239, 240, 242, and ID417) have expression level higher than 0.2 RPM in most of sequenced small RNA libraries (Supplementary Table 8). All five of them are −3p sequences, which are considered as main forms of biologically active miR162 (Kozomara et al., 2019), which indicates that this form is also crucial in yellow lupine. All of them exhibit a similar accumulation profile, with the highest expression in pod walls (PW3), flower pedicels (FPNAB and FPAB), and the lowest in the youngest abscissing and non-abscissing pods (PAB and PNAB) (Figure 6). The miR162 of ID239 not only has the greatest number of annotations but also displays the highest expression.

Analyses of the data for miR162 present in LuluDB indicate that this miRNA can also regulate the expression of *DCL1* in yellow lupine (Supplementary Table 9). All five miRNAs have a long list of targets identified by both degradome and psRNATarget analysis. In each case, we have information about miRNA binding sites, which are located near the center of transcripts in the region devoid of any functional protein domains (Figure 4), similarly to mRNA coding DCL1 in other plant species (Xie et al., 2003; Shao et al., 2015).

It is noteworthy that the download feature is very useful for both analysis and presentation of the data. The downloaded files include information about miRNA ID, its sequence, the list of target genes, and expression of miRNA in different organs. Redirection links to pages for individual targets show that majority of them were identified in degradomes and that they are in fact transcripts annotated as *DCL1*. They are present in both flowers and pods (Supplementary Table 9). Most of them exhibit low level of accumulation, accompanied by high expression of miR162 (ID239), which suggests a negative correlation and tight regulation of *DCL1* mRNA levels by its degradation, or localization of the transcript limited to specific type of cells. Interestingly, sequence miR162-5p (ID183) also has degradome-identified targets in yellow lupine annotated as putative clathrin assembly protein (Supplementary Table 9); however, given the fact that expression levels of this miRNA are very low, its regulatory potential is highly unlikely.

**Figure 7:**
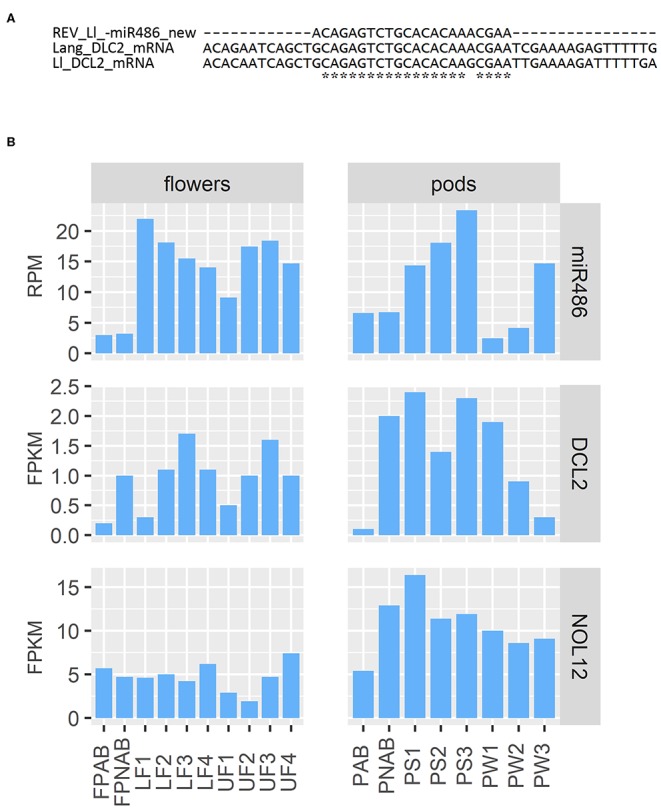
Analysis of miR486. **(A)** Comparison of target sequence for miR486 in DCL2-encoding transcript in yellow and narrow-leaved lupines. **(B)** Expression of miR486 and its selected target genes.

Regarding the already mentioned novel miRNA ID486, which is most likely responsible for regulation of *DCL2* in yellow lupine, there is a long list of targets found by degradome analysis in both flowers and pods in the database (Table 3). Most of them are annotated as *DCL2*; however, some of them encode homologs of Nucleolar protein 12 (NOL12). NOL12 is a protein described, e.g., in humans as a multifunctional protein (Scott et al., 2017): with its ability to bind rRNA, it is required for efficient separation of large and small subunit precursors, and by binding with DNA repair proteins, it is essential for genome integrity. Interestingly, the potential target site for miRNA ID486 is also present within *DCL2* sequence of narrow-leaved lupine (XM_019584571.1) (Figure 7A), which can indicate that this regulator is characteristic for this closely related lupine species. The expression of miRNA ID486 is the highest in LF1 and decreases during flower development, contrary to upper flowers that exhibit the highest level of expression at stage 3 (UF3). In the case of pods, it is strongly accumulated in the oldest seeds (PS3) (Figure 7B). Expression of its target genes is the highest in flowers at stages 3 and 4 independently from their position on inflorescence, as well as in young seeds (PS1), so it is exhibiting a reverse tendency in comparison to miRNA (Figure 7B).

**Table 3:** List of target genes for novel miR486 from *Lupinus luteus*.

**LuluDB transcript**	**Tissue**	**Target sequence**	**Target start position**	**Target end position**	**Regulation type**	**Transcript annotation**
Ll_transcript_256739	Flowers	GCAGAGTCTGCACACAAACGAA	903	924	Cleavage	Endoribonuclease Dicer homolog 2
Ll_transcript_256747	Flowers	GCAGAGTCTGCACACAAGCGAA	903	924	Cleavage	Endoribonuclease Dicer homolog 2
Ll_transcript_256729	Flowers	GCAGAGTCTGCACACAAACGAA	1115	1136	Cleavage	Endoribonuclease Dicer homolog 2
Ll_transcript_256731	Flowers	GCAGAGTCTGCACACAAACGAA	903	924	Cleavage	Endoribonuclease Dicer homolog 2
Ll_transcript_256736	Flowers	GCAGAGTCTGCACACAAACGAA	1115	1136	Cleavage	Endoribonuclease Dicer homolog 2
Ll_transcript_479081	Pods	GCAGAGTCTGCACACAAACGAA	1131	1152	Cleavage	Endoribonuclease Dicer homolog 2
Ll_transcript_479083	Pods	GCAGAGTCTGCACACAAACGAA	1491	1512	Cleavage	Endoribonuclease Dicer homolog 2
Ll_transcript_479084	Pods	GCAGAGTCTGCACACAAACGAA	1020	1041	Cleavage	Endoribonuclease Dicer homolog 2
Ll_transcript_479065	Pods	GCAGAGTCTGCACACAAACGAA	1131	1152	Cleavage	Endoribonuclease Dicer homolog 2
Ll_transcript_479068	Pods	GCAGAGTCTGCACACAAACGAA	919	940	Cleavage	Endoribonuclease Dicer homolog 2
Ll_transcript_256739	Flowers	GCAGAGTCTGCACACAAACGAA	903	924	Cleavage	Endoribonuclease Dicer homolog 2
Ll_transcript_256726	Flowers	GCAGAGTCTGCACACAAACGAA	1115	1136	Cleavage	Endoribonuclease Dicer homolog 2
Ll_transcript_256727	Flowers	GCAGAGTCTGCACACAAACGAA	1115	1136	Cleavage	Endoribonuclease Dicer homolog 2
Ll_transcript_256729	Flowers	GCAGAGTCTGCACACAAACGAA	1115	1136	Cleavage	Endoribonuclease Dicer homolog 2
Ll_transcript_256734	Flowers	GCAGAGTCTGCACACAAACGAA	1115	1136	Cleavage	Endoribonuclease Dicer homolog 2
Ll_transcript_256736	Flowers	GCAGAGTCTGCACACAAACGAA	1115	1136	Cleavage	Endoribonuclease Dicer homolog 2
Ll_transcript_479081	Pods	GCAGAGTCTGCACACAAACGAA	1131	1152	Cleavage	Endoribonuclease Dicer homolog 2
Ll_transcript_479083	Pods	GCAGAGTCTGCACACAAACGAA	1491	1512	Cleavage	Endoribonuclease Dicer homolog 2
Ll_transcript_479084	Pods	GCAGAGTCTGCACACAAACGAA	1020	1041	Cleavage	Endoribonuclease Dicer homolog 2
Ll_transcript_479065	Pods	GCAGAGTCTGCACACAAACGAA	1131	1152	Cleavage	Endoribonuclease Dicer homolog 2
Ll_transcript_479068	Pods	GCAGAGTCTGCACACAAACGAA	919	940	Cleavage	Endoribonuclease Dicer homolog 2
Ll_transcript_219270	Flowers	TCAGGGTCTGCGCGCAAACAAA	2411	2432	Cleavage	Nucleolar protein 12
Ll_transcript_219271	Flowers	TCAGGGTCTGCGCGCAAACAAA	1000	1021	Cleavage	Nucleolar protein 12
Ll_transcript_219276	Flowers	TCAGGGTCTGCGCGCAAACAAA	657	678	Cleavage	Nucleolar protein 12
Ll_transcript_219278	Flowers	TCAGGGTCTGCGCGCAAACAAA	1089	1110	Cleavage	Nucleolar protein 12
Ll_transcript_386361	Pods	TCAGGGTCTGCGCGCAAACAAA	2294	2315	Cleavage	Nucleolar protein 12
Ll_transcript_386366	Pods	TCAGGGTCTGCGCGCAAACAAA	2412	2433	Cleavage	Nucleolar protein 12
Ll_transcript_386378	Pods	TCAGGGTCTGCGCGCAAACAAA	1255	1276	Cleavage	Nucleolar protein 12
Ll_transcript_386378	Pods	TCAGGGTCTGCGCGCAAACAAA	1255	1276	Cleavage	Nucleolar protein 12

Homologs of both miR162 as well as new miR486 do not show differential expression in pedicels of abscissing and non-abscissing flowers similarly to abscissing and non-abscissing pods, which can indicate that they are not directly linked to the generative organ abscission process in yellow lupine. However, changes in their accumulation during the development suggest that they regulate the sRNA biogenesis depending on the stage of development in both flowers and pods.

### Other Examples and Suggestions for the Use of Data Contained in the LuluDB

The data present in the database was already used to identify new mechanisms for regulating gene expression by sRNA in yellow lupine; e.g., we described the involvement of sRNAs in *L. luteus* flower development including new miRNAs and the new siRNA (siR240), which, together with the conserved tasiR-ARF, may trigger cleavage of the *TAS3* transcript (Glazinska et al., 2019). Data present in the database were also used to perform analyses published by Shi and coworkers (Shi et al., 2019), where the authors identified yellow lupine *IDA* (*INFLORESCENCE DEFICIENT IN ABSCISSION*), *IDL* (*IDA-LIKE*), and *HSL* (*HAESA*) transcripts, and compared their expression with their homologs in other plant species. The authors proved that the lupine gene most similar to *AtIDA* (*LlIDA*) plays the most important role in lupine AZs compared to the *LlIDL* genes. This work indicates that the mechanism of the generative organ abscission in lupine and *A. thaliana* has common features. These papers were published before the LuluDB was made public and we believe that publication of an article on our database will encourage more researchers to use it for their own purposes.

To date, the LuluDB is profiled to provide information about the regulation of transcripts by miRNA and siRNA, confirmed by degradome analysis. However, it also contains a category of “lncRNA,” which was not explored by our group, and gives the opportunity to perform preliminary *in silico* analyses of this kind of regulatory factors. Additionally, a significant amount of transcripts are not annotated to any database. The algorithms for protein–protein and protein–ligand docking are constantly being improved, giving the opportunity to specify the function of chosen protein basing solely on its sequence. Today, guessing that the function of all the unannotated protein-coding transcripts would be impossible—this method is time- and memory-consuming. However, in the near future, computers are going to be better equipped and algorithms are going to be faster, so probably this kind of analyses will be performed.

## Conclusion

**Figure 8:**
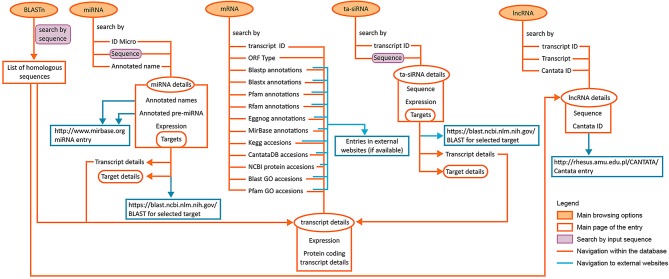
Diagram depicting the possible ways to access and explore the database.

The presented LuluDB database has been equipped with user-friendly and intuitive tools for searching and investigating our NGS data, including more advanced bioinformatics. Figure 8 depicts possible approaches to explore the database and navigate its components, as well as their interlinked sections. The presented case study visualizes how the use of LuluDB database improves the process of analysis of both miRNAs and their target genes involved in regulation of growth or abscission of generative organs in yellow lupine, and allows for identification of homologs of selected genes and evolutionary analyses. The database can be used as a starting point for different types of research concerning protein-coding RNAs and ncRNAs not only for yellow lupine but also for other plant species.

## Materials and Methods

### Plant Material

Yellow lupine plants used for RNA extraction were cultivated in Nicolaus Copernicus University's experimental field in Piwnice near Torun (Poland, 53°05′42.0″N 18°33′24.6″E), as described in detail in Glazinska et al. (2019). The flowers were separated based on their developmental stage and position on raceme, as described previously in Glazinska et al. (2019). Additionally, flower pedicles from abscissing (FPAB) and non-abscissing flowers (FPNAB) were collected. Then, pods in different stages of development were also harvested and separated into pod walls (PW) and seeds (PS). Only the pods from the lowest whorl were collected, based on the fact that most of the pods set at higher whorls undergo abscission at early stage of development (van Steveninck, 1957). In addition, entire small abscissing pods (PAB) as well as non-abscissing pods of the same length (PNAB) were collected as a whole.

### RNA Isolation, Library Construction, and NGS of Small RNA, Transcriptome, and Degradome

RNA isolation from all of the collected samples was carried out using miRNeasy Mini Kit (Qiagen, Venlo, the Netherlands) with on-column DNA digestion with the RNase-Free DNase Set (Qiagen, Venlo, the Netherlands), as described in detail in Glazinska et al. (2019). After passing all quality and quantity checks (as described in Glazinska et al., 2019), total RNA was used for preparation of small RNA libraries using NEBNext Multiplex Small RNA Library Prep kit for Illumina (New England Biolabs, Ipswich, MA, USA) and subsequently sequenced on the HiSeq4000 platform (Illumina, San Diego, CA, USA) in the 50 single-end mode.

Similarly isolated total RNA was used to create transcript libraries using the NEBNext Ultra Directional RNA Library Prep Kit for Illumina (New England Biolabs, Ipswich, MA, USA) and sequenced on the HiSeq4000 platform in the 100 paired-end mode as described in detail in Glazinska et al. (2019).

Degradomes were obtained using total RNA pooled from samples UF3/LF3 and PW3/PS3 to meet the amount of material required for sequencing. The protocol for degradome library preparation and detailed information can be found in Glazinska et al. (2019). The small RNA libraries were created from two biological replicates, transcriptomes from three pooled replicates, and degradomes from pooled samples of LF3–UF3 and PS3–PW3; thus, in total, we have sequenced 32 small RNA, 16 transcriptome, and 2 degradome libraries.

### RNA-Seq: *de novo* Transcriptome Assembly and Transcript Expression Estimation

The *de novo* transcriptome assembly was performed on RNA-Seq data using Trinity v 2.4.0 (https://github.com/trinityrnaseq/trinityrnaseq/releases) with default settings as described in Glazinska et al. (2017). The transcriptome assembly for flowers and pods was carried out separately. Therefore, to make this division clear, identified transcripts from flowers have an additional letter “F” as a prefix (e.g., FTRINITY_DN53848_c2_g1_i5). Estimation of expression level on both the unigene and isoform level was reported in FPKM and was done using RSEM (Haas et al., 2013) as described in Glazinska et al. (2017).

### RNA-Seq: Annotating the Transcriptomes

Annotation of transcriptomes was performed with Trinotate (v 3.0.2). BLASTX with “max_target_seqs 1” option was used to identify the sequence similarity between lupine transcripts and proteins annotated in Swiss-Prot, a non-redundant and manually curated dataset from the UniProt database. Open reading frames were predicted with TransDecoder (v 5.0.1) (Haas et al., 2013) in order to scan inferred protein sequences against Swiss-Prot using BLASTP (with “-max_target_seqs 1” option). hmmscan (hmmer.org) with default settings was used to identify protein domains based on the similarity to Pfam database records (http://pfam.xfam.org/). All of the previously mentioned results were loaded into an SQLite database built by Trinotate and used to generate the final report. Another approach for annotating the transcriptomes was also applied using sole BLAST searches against public databases, which included BLASTN against RFAM (no *E*-value threshold), miRBase (*E*-value threshold of 1e^−5^), and *G. max* lncRNAs from CantataDB (Szcześniak et al., 2016) (*E*-value threshold of 1e^−5^). Additionally, the BLASTX search against the NCBI protein database (nr) for *Fabaceae* (no *E*-value threshold) was performed. The results were then parsed to obtain the best hits per transcript based on the alignment score value.

### Identification of Small ncRNAs and Their Target Genes

To identify phylogenetically conserved mature miRNAs with sequences and lengths identical to known plant miRNAs, we searched miRBase for similarity at the mature miRNA level. Short reads from RNA-Seq were compared against mature miRNAs from miRBase (Kozomara et al., 2019). The comparison was performed with Bowtie (Langmead, 2010), allowing for no mismatches. To predict potential novel miRNAs, we applied ShortStack (Axtell, 2013b) with default settings as described in Glazinska et al. (2019). ShortStack (Axtell, 2013b) was also used to identify small RNAs that were being cut in phase from longer precursors (phased siRNAs) (Glazinska et al., 2019). The top 200 candidates were selected from each sample, based on the phased score value provided by ShortStack. Finally, lists of such sRNAs from all samples were merged into a single dataset of non-redundant phased siRNAs. The expression of sncRNA was presented in RPM units. Each discovered miRNA received an identification ID number. MiRNAs identified using ShortStack and not showing sequence similarity with miRBase were given numbers from 457 up and have the annotation “none” in the database. Target genes for identified small RNAs were estimated based on degradome data, or using psRNATarget tool as described in Glazinska et al. (2019). The miRNA targets were searched among assembled transcriptomes, separately for flowers and for pods. The same analysis was done for siRNAs.

### Expression Analysis With RT-qPCR

MiRNA and siRNA expression was analyzed using the Stem Loop RT-qPCR technique according to Glazinska et al. (2019). Expression of protein coding transcripts was measured as in Glazinska et al. (2017). Each experiment consisted of three biological and technical replicates. The relative expression levels were calculated using the 2^−ΔΔCt^ method, and the data were normalized to the CT values for the *LlActin4* reference gene (according to Glazinska et al., 2017). All primer sequences are listed in Supplementary Table 10. To assess the linearity of relationship and correlation strength between sRNA-Seq or RNA-Seq and qPCR data, we have first log-transformed the data and calculated *R*^2^ and Spearman's rank correlation coefficient (ρ), respectively, using R packages: dplyr, ggpubr, and Hmisc.

### Data Submission to Sequence Read Archive (NCBI)

The RNA-Seq data and small RNA-Seq data have been uploaded to SRA database and are available under BioProject ID PRJNA419564 and Submission ID SUB3230840.

### Database Implementation and Testing

LuluDB was developed using Hypertext Markup Language (HTML), Sassy Cascaded Style Sheets (SCSS), Cascading Style Sheets (CSS), PHP 5.6, Yii 2.0 PHP framework (https://www.yiiframework.com/), MySQL 5.5, JavaScript, jQuery 3.2.1 (https://jquery.com/), MSAViewer (Yachdav et al., 2016), and Bootstrap 3.3.7 framework (https://getbootstrap.com/). NCBI BLAST+ 2.7.1 (Camacho et al., 2009) was used as a local alignment search tool. Database can be run and has been tested on most currently widely used web browsers regardless of operating system, including Firefox Web Browser, Safari, Google Chrome, and Opera. Responsive web design was applied to ensure that the database will be properly displayed on mobile devices.

## Data Availability Statement

All datasets analyzed for this study are included either in the article/Supplementary Material or in the database at http://luluseqdb.umk.pl/basic/web/index.php. The RNA-Seq data and small RNA-Seq data have been uploaded to SRA database and are available under BioProject ID PRJNA419564 and Submission ID SUB3230840.

## Author Contributions

PG: conceptualization, funding acquisition, supervision, and writing—review and editing. PG, MW, and JK: data curation. PG, MK, and WG: investigation, visualization, and writing—original draft. JK and MW: software.

## Conflict of Interest

The authors declare that the research was conducted in the absence of any commercial or financial relationships that could be construed as a potential conflict of interest.

## Supplementary Material

Supplementary Figure 1 Alignment of sequences of corresponding DCL1-coding transcripts expressed in flowers and pods.

Supplementary Figure 2 Screenshot of LuluDB page concerning: **(A)** phased-siRNA, **(B)** long non-coding RNA.

Supplementary Figure 3 Screenshot of LuluDB page concerning protein-coding RNA sequence.

Supplementary Figure 4 Juxtaposition of NGS and qPCR expression levels of eight transcripts used for validation. Homologs of the same transcript found in flowers and pods are shown separately.

Supplementary Table 1 Details of data deposition in NCBI SRA.

Supplementary Table 2 A list of DCL sequences from *Arabidopsis thaliana* and selected *Fabaceae* plants used for identification of homologs in *L. luteus* and in phylogenetic analyses.

Supplementary Table 3 Results of LuluDB search by built-in BLASTn using *A. thaliana DCL1* CDS sequence as query.

Supplementary Table 4 Results of LuluDB search by built-in BLASTn using *A. thaliana DCL2* CDS sequence as query.

Supplementary Table 5 Results of LuluDB search by built-in BLASTn using *A. thaliana DCL3* CDS sequence as query.

Supplementary Table 6 Results of LuluDB search by built-in BLASTn using *A. thaliana DCL4* CDS sequence as query.

Supplementary Table 7 List of regulatory sequences identified within 5′UTRs of *L. luteus* mRNAs coding for DCL1, 3 and 4 using PlantCare search.

Supplementary Table 8 A detailed list of miRNAs in LuluDB annotated as miR162.

Supplementary Table 9 List of target transcripts for members of *MIR162* family deposited in LuluDB.

Supplementary Table 10 List of primers and UPL probes used for RT-qPCR reaction.
