# Supplementary figures and images for: LuluDB—The Database Created Based on Small RNA, Transcriptome, and Degradome Sequencing Shows the Wide Landscape of Non-coding and Coding RNA in Yellow Lupine (Lupinus luteus L.) Flowers and Pods
Source: Front Genet. 2020 May 15;11:455. doi: 10.3389/fgene.2020.00455 (PMC7242762; doi:10.3389/fgene.2020.00455)

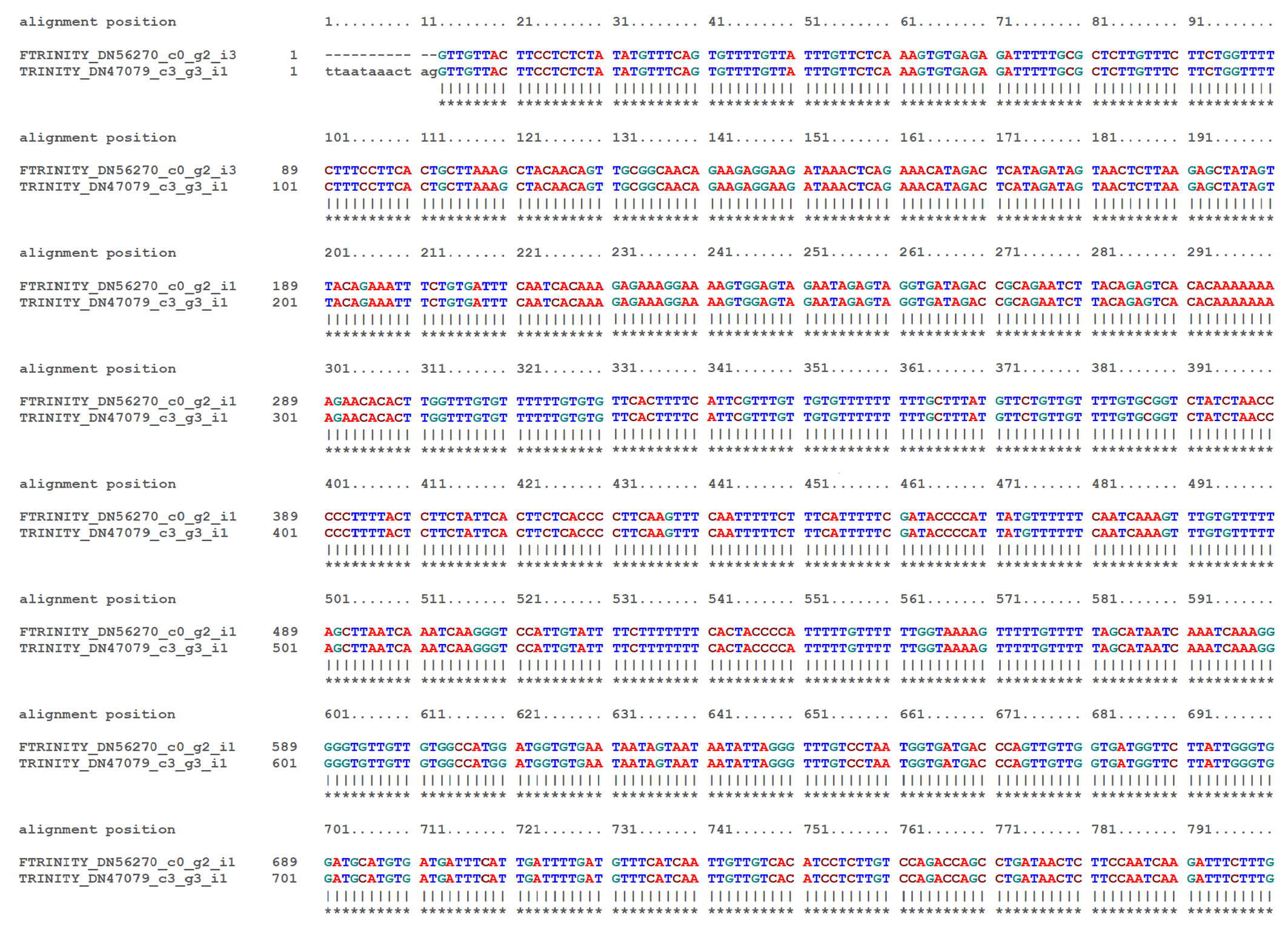

Supplement: Supplementary Figure 1 — Alignment of sequences of corresponding DCL1-coding transcripts expressed in flowers and pods. [file Image_1.JPEG]

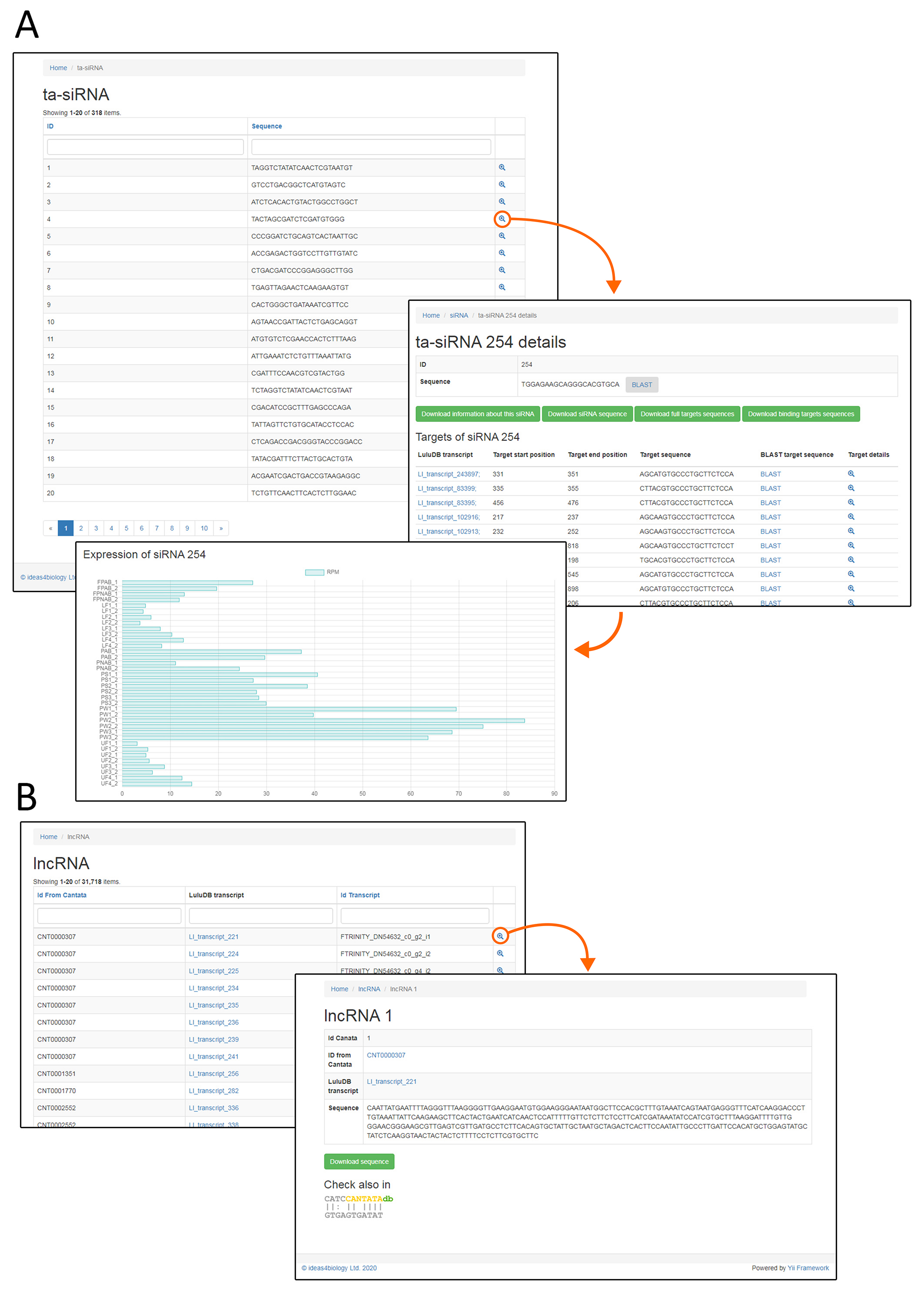

Supplement: Supplementary Figure 2 — Screenshot of LuluDB page concerning: (A) phased-siRNA, (B) long non-coding RNA. [file Image_2.JPEG]

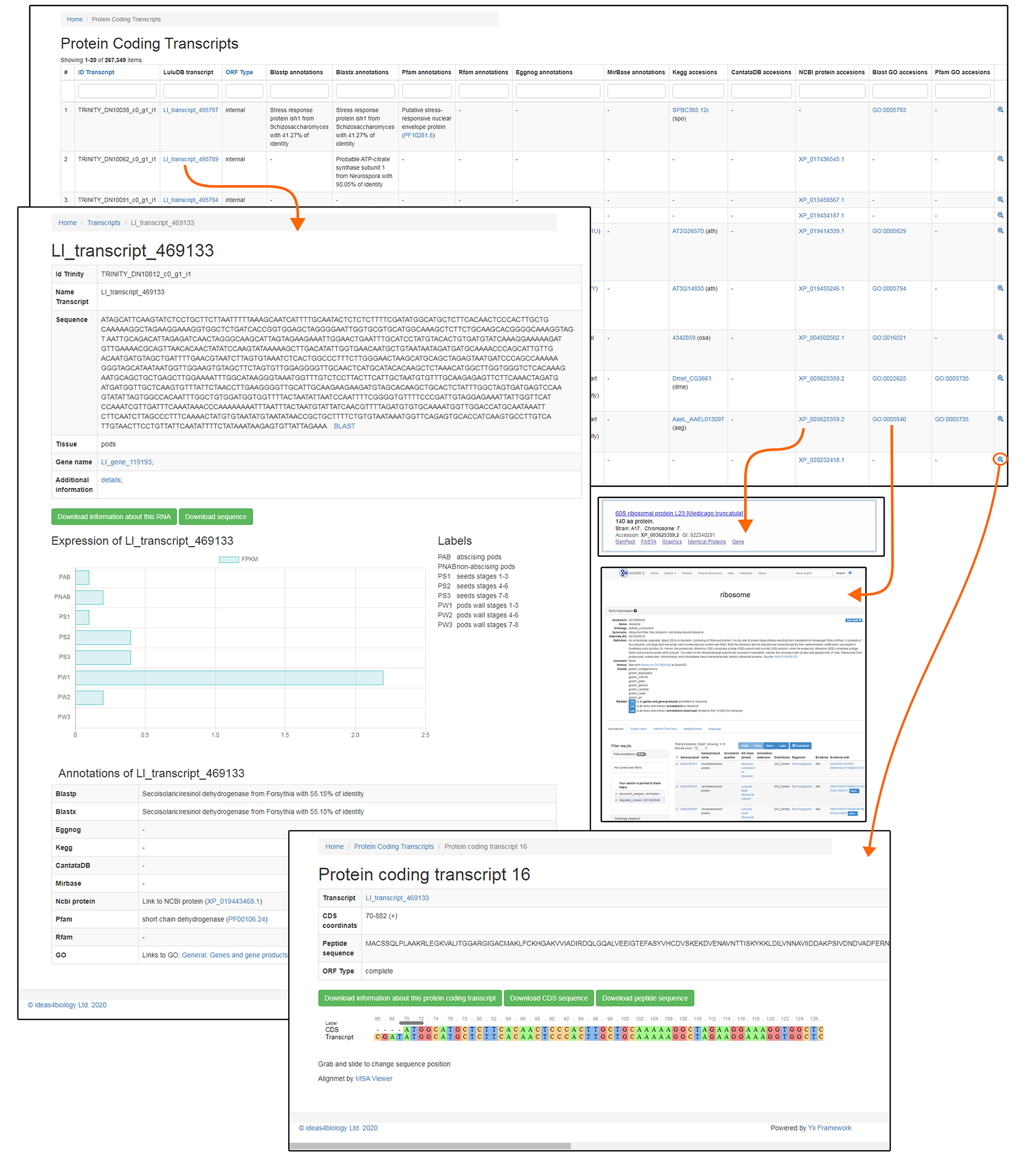

Supplement: Supplementary Figure 3 — Screenshot of LuluDB page concerning protein-coding RNA sequence. [file Image_3.JPEG]

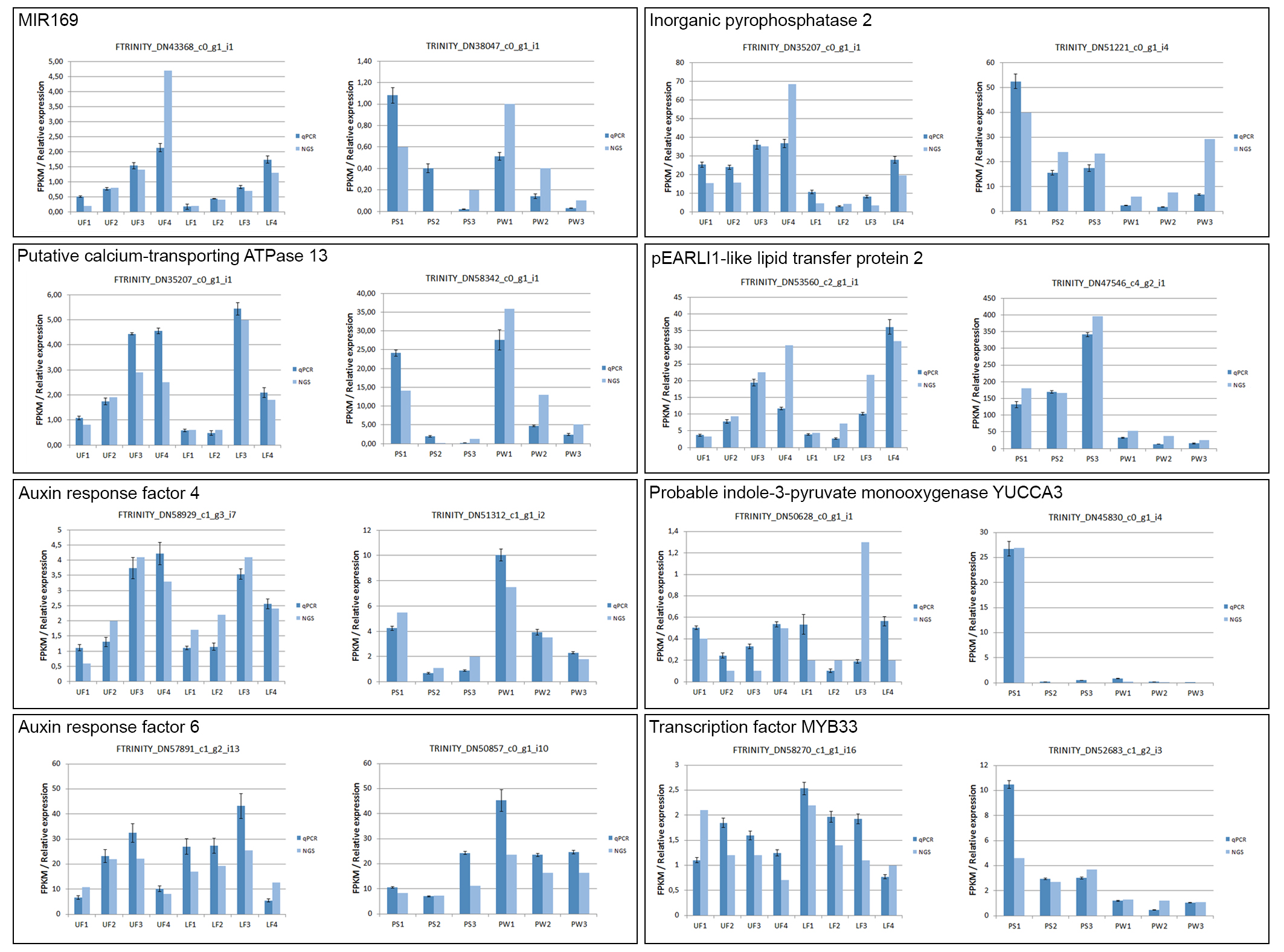

Supplement: Supplementary Figure 4 — Juxtaposition of NGS and qPCR expression levels of eight transcripts used for validation. Homologs of the same transcript found in flowers and pods are shown separately. [file Image_4.JPEG]
